# Posttraumatic Stress Disorder Among Adults in Communities With Mass Violence Incidents

**DOI:** 10.1001/jamanetworkopen.2024.23539

**Published:** 2024-07-26

**Authors:** Angela D. Moreland, Caitlin Rancher, Faraday Davies, Jamison Bottomley, Sandro Galea, Mohammed Abba-Aji, Salma M. Abdalla, Michael G. Schmidt, John E. Vena, Dean G. Kilpatrick

**Affiliations:** 1 Department of Psychiatry and Behavioral Sciences, Medical University of South Carolina, Charleston; 2 School of Public Health, Boston University, Boston, Massachusetts; 3 Department of Microbiology and Immunology, Medical University of South Carolina, Charleston; 4 Department of Public Health Sciences, Medical University of South Carolina, Charleston

## Abstract

**Importance:**

Mass violence incidents (MVIs) are prevalent in the US and can have profound and long-lasting psychological consequences on direct survivors, but their outcomes among the broader communities where the MVI occurred are unknown.

**Objective:**

To investigate the prevalence of and factors associated with past-year and current posttraumatic stress disorder (PTSD) among adults in communities that experienced an MVI.

**Design, Setting, and Participants:**

This cross-sectional survey was completed between February and September 2020 with a household probability sample of adults from 6 communities that had experienced an MVI between 2015 and 2019: Dayton, Ohio; El Paso, Texas; Parkland, Florida; Pittsburgh, Pennsylvania; San Bernadino, California; and Virginia Beach, Virginia. Address-based sampling was used to identify randomly selected households, mail invitations, and select 1 adult per household to complete a self-administered paper or online survey. Data analysis was performed from September to November 2023.

**Main Outcomes and Measures:**

The primary outcome was presumptive diagnostic-level past-year PTSD and current (past month) PTSD determined using American Psychiatric Association *Diagnostic and Statistical Manual of Mental Disorders* (Fifth Edition) (*DSM-5*) PTSD criteria.

**Results:**

A total of 6867 adults aged 18 years or older accessed the website with a description of the study and consent materials. Of these, 5991 (87.2%) agreed to participate and completed the survey, 343 (6.3%) partially completed the survey, and 443 (6.5%) did not meet eligibility criteria or refused to participate. Most of the 5991 respondents were female (3825 individuals [53.5%]) and had a mean (SD) age of 45.56 (17.58) years. A total of 1261 of 5931 individuals (21.0%) reported high exposure to the MVI (either they or a close friend and/or family member was on site during the shooting). Nearly one-quarter (23.7%; 1417 of 5977 participants reporting PTSD) met presumptive *DSM-5* diagnostic criteria for past-year PTSD, with 8.9% (530 participants) meeting the criteria for current PTSD. Regression analyses found that being female (odds ratio, 2.32; 95% CI, 2.01-2.68) and having a history of both physical or sexual assault and other potentially traumatic events (odds ratio, 9.68; 95% CI, 7.48-12.52) were associated with the greatest risk of past-year PTSD.

**Conclusions and Relevance:**

In this survey study of 5991 participants, presumptive PTSD was quite prevalent long after the MVI among adults in communities that have experienced an MVI, suggesting that MVIs have persistent and pervasive public health impacts on communities, particularly among those with prior exposure to physical or sexual assault and other potentially traumatic events. Focusing exclusively on direct exposure to MVIs is not sufficient. Incorporating these findings into screening should improve efforts to identify the individuals most in need of prevention or mental health service after MVIs.

## Key Points

**Question:**

Do adults in communities that experienced a mass violence incident (MVI) have higher prevalence of and factors associated with past-year and current posttraumatic stress disorder (PTSD)?

**Findings:**

In this cross-sectional survey of a probability sample of 5991 adults living in communities that had experienced an MVI, there was a high prevalence of past-year (23.7%) and current (8.9%) PTSD. Being female, having a history of physical or sexual assault, and having a history of other potentially traumatic events were associated with the greatest risk of PTSD.

**Meaning:**

These findings suggest that the outcomes of MVIs in communities extend beyond direct survivors, including persistent PTSD in many adults, and are exacerbated by exposure to prior traumatic events; thus, screening efforts for mental health services after MVIs should not focus exclusively on those directly exposed to MVIs.

## Teaser

This survey study examines the prevalence of and factors associated with past-year and current posttraumatic stress disorder among adults in 6 US communities that experienced a mass violence incident.

## Introduction

Mass violence incidents (MVIs) have profound and long-lasting psychological and behavioral effects on survivors, including posttraumatic stress disorder (PTSD).^1,2,3^ The impact of MVIs may extend far beyond directly affected survivors and their families, because ripple effects can extend to the entire affected community. Specifically, studies following the attacks of September 11, 2001 (9/11), indicated that members of the community reported high levels of PTSD following the attack. Although individuals with highest exposure to the MVI had the highest rates of PTSD, individuals with indirect exposure also reported substantial mental health concerns.^4,5^

The majority of findings on MVI impact on broad communities focus on a specific community or event, most commonly the 9/11 terrorist attacks^5^ or school campus shootings.^6^ Less is known about the mental health outcomes, especially rates of PTSD, on entire communities affected by MVIs. Previous reviews suggest that, among individuals exposed to MVI, rates of PTSD vary greatly according to demographic characteristics, level of exposure, other mental health and pre-MVI characteristics, and latency since the MVI.^1^ Specifically, higher rates of PTSD following MVI exposure were seen among female individuals, low-income or unemployed individuals, and those with lower education.^1^ Factors specific to the MVI, including direct exposure and shorter time since the event, were also associated with PTSD.^1,7^ Prior exposure to potentially traumatic events (PTEs) has also been found to increase rates of PTSD among individuals exposed to MVIs.^8,9^ Several studies show that prior exposure to PTEs involving physical or sexual assault in particular is an important risk factor for PTSD following natural disasters,^10^ exposure to toxic chemicals,^11^ and new incidents of violent crime.^12^ This suggests it is important to examine the extent to which exposure to physical or sexual assault is associated with increased risk of PTSD following MVIs, beyond that of other PTEs.

Given the increasing rate of MVIs in the US over the past decade^13^ and the major consequences associated with high rates of PTSD among individuals, it is imperative to further examine rates of PTSD among individuals residing in broad communities impacted by mass violence. Thus, this article reports survey results from a household probability sample of adults from 6 communities impacted by MVIs from 2015 to 2019. Objectives included assessing rates of PTSD (past-year and current) following MVIs across demographic characteristics (age, race and ethnicity, gender, income, and education), risk factors unrelated to the MVI (exposure to physical or sexual assault PTEs, other PTEs, and low social support), and risk factors specific to the MVI (number of months since the MVI and level of exposure). We hypothesized that individuals with prior exposure to PTEs, low social support, and higher levels of exposure to MVI would report more symptoms of PTSD. Results of this study have potential public health implications, given the substantial disease burden associated with PTSD and related mental health concerns,^14^ for response and treatment needs of communities following MVIs.

## Methods

### Data Collection and Sample

This report follows the 11 transparency initiative disclosure elements outlined by American Association for Public Opinion Research (AAPOR) reporting guidelines.^15^ Data were collected sequentially between February and September 2020, with data collection for each site lasting approximately 2 months, from a household probability sample of adults living in 6 communities that experienced an MVI between 2015 and 2019 (Dayton, Ohio; El Paso, Texas; Parkland, Florida; Pittsburgh, Pennsylvania; San Bernadino, California; and Virginia Beach, Virginia) using address-based sampling. Letters with a brief description of the study were sent to randomly selected households within specified geographic areas. One adult per household was randomly selected and asked to complete an online or mail survey about their experiences with and responses to the MVI. Participants gave electronic or written informed consent, and the Institutional Review Board for Human Research at the Medical University of South Carolina approved the study. The participant enrollment flowchart is shown in eFigure 1 in Supplement 1. Several procedures encouraged participation and willingness to provide candid responses about sensitive matters. Names and contact information were not attached to survey responses. Respondents were informed that we had a Privacy Certificate from the Department of Justice that provides total confidentiality, including protection from disclosure via subpoena in federal or state court. Respondents who completed the survey were reimbursed $15. Further details on sampling, data collection, and key measures are provided in eAppendixes 1 and 2 and eTable 1 in Supplement 1.

### Measures

#### Prevalence of Past-Year and Current PTSD

PTSD was measured using the National Stressful Events Survey PTSD Module developed in conjunction with the *Diagnostic and Statistical Manual of Mental Disorders* (Fifth Edition) (*DSM-5*) PTSD Workgroup^16^ (eAppendix 1 in Supplement 1). Participants completed 20 items assessing each *DSM-5* PTSD symptom, as well as 2 items assessing whether symptoms have resulted in substantial distress or impairment. This measure further assesses how recently diagnostic criteria have been met (ie, within the past year or within the past month). Responses were aggregated and coded to determine presumptive diagnostic-level PTSD for both past-year and current PTSD (see eAppendix 1 in Supplement 1 for more details). For the present sample, α = .93.

#### Potentially Traumatic Events

Participants completed an 11-item measure of exposure to PTEs from the National Stressful Events Survey PTSD Module that included combat exposure, serious accidents, life-threatening illnesses, and physical or sexual assault (eg, “Has anyone ever used physical force or threats of force to make you have some type of unwanted sexual contact?”). Responses to each PTE were no (0) or yes (1). Responses were coded to create 4 groups of participants: those who had experienced (1) physical and sexual assault PTEs, (2) other PTEs, (3) both physical and sexual assault PTEs and other PTEs, and (4) no history of any PTEs.

#### Social Support

Participants completed 5 items from the Medical Outcomes Study module assessing the social support they received in the past 6 months (eg, “How often was someone available to confide in or talk about your problems?”)^17^ (eAppendix 1 in Supplement 1). Responses had a 4-point scale, ranging from 1 (none of the time) to 4 (all of the time). Higher scores on this measure (range, 5-20) have been associated with lower levels of psychological distress, with a score of 15 or lower indicating low social support. Numerous studies have used this measure, including studies of the 9/11 terrorist attacks^4^ and hurricanes in Florida.^16,18^ In the present sample, α = .91.

#### Latency

The length of time since the MVI was calculated by subtracting the survey completion date from the date of the MVI. Latency is reported in months.

#### Exposure to the MVI

Participants completed 12 items assessing their level of exposure to the MVI specific to their community (eg, “You personally were shopping or working at the Walmart Supercenter near the Cielo Visto Mall in El Paso, Texas when the shooting happened,” or “You were the parent, guardian, other relative, or close friend of a student or worker who was at Marjory Stoneman Douglas High School the day of the shooting”). Responses to items were coded to indicate whether participants had high levels of exposure (0 = no, 1 = yes) to the MVI defined as either they or a close friend and/or family member was on site during the shooting.

#### Demographic Characteristics

Participants completed self-report items assessing their demographic characteristics (eg, gender, race, and ethnicity). Data on race and ethnicity are included in this study to provide valuable information regarding potential difference in MVI impact among subgroups.

### Statistical Analysis

Data analysis was performed from September to November 2023. To examine our hypotheses, we conducted univariate comparisons to examine between-group differences in the prevalence of past-year and current PTSD across demographic characteristics (age, race and ethnicity, gender, income, and education), risk factors unrelated to the MVI (exposure to PTEs and low social support), and risk factors specific to the MVI (short latency since the MVI and high levels of exposure). The threshold for statistical significance was 2-sided. Next, we simultaneously entered the demographic characteristics, risk factors unrelated to the MVI, and risk factors specific to the MVI into logistic regression analyses to assess which variables were uniquely associated with PTSD after accounting for the other variables in the model. We ran separate models for past-year and current PTSD.

All analyses were weighted to adjust for potential nonresponse bias by first weighting to adjust for household size and likelihood of household nonresponse and then using iterative proportional fitting to align with population benchmarks on gender, age, education, race, ethnicity, and stratum. The weighting parameters were based on the US Census Bureau’s 2018 American Community Survey 5-year estimates. The weighted demographics matched or were very close to all Census benchmarks for each community. See eAppendix 2 and eTable 2 in Supplement 1 for more detail.

Analyses were conducted using SPSS statistical software version 28 (IBM). A missing values analysis indicated all items had less than 5% missingness, and preliminary examination of our regression model indicated 10% missing cases; therefore, we conducted available case analysis. A sensitivity power analysis indicated that with 10 independent variables, α = .05, and our most conservative sample of 5402 participants, power exceeded 0.99 to detect small effect sizes (*f*^2^ = 0.02).

## Results

### Descriptive Statistics

Invitations were mailed to 110 289 addresses in 6 communities that had experienced an MVI. A total of 6867 adults aged 18 years or older accessed the website with a description of the study and consent materials. Of these, 5991 (87.2%) agreed to participate and completed the survey (response rate, 5.4%), 343 (6.3%) partially completed the survey, and 443 (6.5%) did not meet eligibility criteria or refused to participate. Among the final sample of 5991 respondents, most were female (3825 individuals [53.5%]) and had a mean (SD) age of 45.56 (17.58) years. Demographic characteristics are reported in Table 1. Most participants self-identified as White (70.6%; 4528 of 5880 reporting race) and non-Hispanic (74.4%; 4623 of 5951 reporting ethnicity); 63 participants (1.0%) were American Indian or Alaska Native, 231 (4.1%) were Asian, 617 (16.5%) were Black or African American, 1328 (25.6%) were Hispanic, and 441 (7.8%) identified as other races and ethnicities (eg, biracial, Jamaican, Caribbean, Taino, North African, Middle Eastern, or Jewish). Latency, or time since the MVI, ranged from 8 to 56 months (mean [SD], 18.94 [11.61] months). Less than 3.0% of participants (160 participants) reported they were personally on site and 19.0% (1136 participants) reported a close friend and/or family member was on site at the MVI, resulting in 21.0% of participants (1261 of 5931 reporting exposure) with high levels of exposure to the MVI. Most participants experienced at least 1 PTE, with 4.8% (283 of 5976 participants reporting PTEs) reporting physical or sexual assault alone, 38.7% (2406 participants) reporting other PTEs, and 37.9% (2240 participants) reporting both physical or sexual assault and other PTEs. Only 18.4% (1046 participants) reported no history of any PTEs. Most participants (56.0%; 3273 of 5843 participants reporting social support) reported low social support (defined as scores ≤15).

**Table 1.** Demographic Characteristics of Survey Respondents

Characteristic	Respondents, No. (unweighted %) [weighted %] (N = 5991)^a^
Age, mean (SD), y	45.56 (17.58)
Latency, mean (SD), mo since mass violence incident	18.94 (11.61)
Social support score, mean (SD)	14.31 (4.63)
Race	
American Indian or Alaska Native	63 (1.1) [1.0]
Asian	231 (3.9) [4.1]
Black or African American	617 (10.5) [16.5]
White	4528 (77.0) [70.6]
Other^b^	441 (7.5) [7.8]
Ethnicity	
Hispanic	1328 (22.3) [25.6]
Non-Hispanic	4623 (77.7) [74.4]
Gender	
Male	2129 (35.5) [46.5]
Female	3825 (64.2) [53.5]
Annual household income, $	
<25 000	1059 (18.6) [26.1]
25 000-49 999	1167 (20.5) [24.8]
50 000-74 999	980 (17.2) [17.2]
75 000-99 999	755 (13.3) [11.2]
≥100 000	1735 (30.5) [20.8]
Education	
High school graduate or less	889 (14.9) [35.5]
Some college or technical training	1768 (29.6) [33.6]
College graduate	1639 (27.5) [19.3]
Graduate work	1671 (28.0) [11.5]
PTEs	
Physical or sexual assault	283 (4.7) [4.8]
Other PTEs	2406 (40.2) [38.7]
Both physical or sexual assault and other PTEs	2240 (37.4) [37.9]
No history of PTE	1046 (17.5) [18.4]
Exposure to mass violence incident	
Yes	1261 (21.0) [18.5]
No	4670 (78.7) [81.5]

Abbreviation: PTE, potentially traumatic event.

^a^ Data are reported using available case analyses.

^b^ Other racial subcategories included participants who reported they were biracial, Jamaican, Caribbean, Taino, North African, Middle Eastern, or Jewish.

### Univariate Comparisons of PTSD Prevalence

Nearly one-quarter of participants (23.7%; 1417 of 5977 participants reporting PTSD) met presumptive *DSM-5* criteria for past-year PTSD, and 8.9% (530 participants) met criteria for current PTSD. Prevalence of PTSD across each community is presented in eFigure 2 in Supplement 1. Correlations among study variables are presented in Table 2. Differences in demographic characteristics across PTSD prevalence are presented in Table 3. See additional descriptions of PTSD prevalence across variables in eTable 3 in Supplement 1. Among the demographic characteristics, being younger, being female, and reporting lower annual household income and educational attainment were associated with both past-year and current PTSD prevalence. Among the risk factors unrelated to the MVI, exposure to both physical or sexual assault and other PTEs and low social support were also associated with increased risk for past-year and current PTSD. Participants reporting high levels of MVI exposure were also more likely to experience past-year and current PTSD. Shorter latency since the MVI was only associated with past-year PTSD.

**Table 2.** Correlations Among Study Variables^a^

Variable	1	2	3	4	5	6	7	8	9	10	11	12
1. Past-year PTSD (0 = no, 1 = yes)	NA	NA	NA	NA	NA	NA	NA	NA	NA	NA	NA	NA
2. Current PTSD (0 = no, 1 = yes)	.56^b^	NA	NA	NA	NA	NA	NA	NA	NA	NA	NA	NA
3. Age	−.18^b^	−.11^b^	NA	NA	NA	NA	NA	NA	NA	NA	NA	NA
4. Race (1 = White, 0 = non-White)	.01	−.03^c^	.03^c^	NA	NA	NA	NA	NA	NA	NA	NA	NA
5. Ethnicity (1 = Hispanic, 2 = non-Hispanic)	.01	−.00	.10^b^	.05^b^	NA	NA	NA	NA	NA	NA	NA	NA
6. Gender (0 = male, 1 = female)	.17^a^	.11^b^	−.02	.02	.01	NA	NA	NA	NA	NA	NA	NA
7. Income	−.09^b^	−.09^b^	.02	.21^b^	.17^b^	−.09^b^	NA	NA	NA	NA	NA	NA
8. Education	−.01	−.05^b^	−.13^b^	.13^b^	.12^b^	−.04^d^	.42^b^	NA	NA	NA	NA	NA
9. Physical or sexual assault (0 = no, 1 = yes)	.30^b^	.23^b^	.03	−.07^b^	.00	.06^b^	−.11^b^	−.05^b^	NA	NA	NA	NA
10. Other potentially traumatic events (0 = no, 1 = yes)	.16^b^	.14^b^	.06^b^	.07^b^	.03^c^	−.05^b^	.14^b^	.07^b^	.69^b^	NA	NA	NA
11. Social support	−.17^b^	−.14^b^	−.05^b^	.09^b^	.14^b^	.00	.23^b^	.16^b^	−.11^b^	−.12^b^	NA	NA
12. Latency (mo since MVI)	−.03^c^	−.01	−.01	−.09^b^	−.09^b^	.01	.05^b^	.02	−.01	−.00	−.03^c^	NA
13. Exposure to MVI (0 = no, 1 = yes)	.10^b^	.10^b^	−.01	−.03^c^	−.05^b^	.06^b^	.05^b^	.01	.05^b^	.04^d^	.00	.07^b^

Abbreviations: MVI, mass violence incident; NA, not applicable; PTSD, posttraumatic stress disorder.

^a^ Weighted comparisons are reported using available case analyses. Phi coefficients were calculated for relationships between dichotomous variables. Point-biserial correlations were calculated between continuous variables and dichotomous variables.

^b^ *P* < .001.

^c^ *P* < .05.

^d^ *P* < .01.

**Table 3.** Prevalence of Past-Year and Current PTSD Across Study Variables

Variable	Participants, weighted % (observed No.)			
	Past-year PTSD		Current PTSD	
	Yes (n = 1417)	No (n = 4560)	Yes (n = 530)	No (n = 5447)
Age, mean (SD), y	39.89 (15.18)	47.34 (17.90)	39.41 (14.82)	46.14 (17.71)
Latency, mean (SD), mo since MVI	18.27 (11.24)	19.15 (11.71)	18.72 (12.16)	18.96 (11.55)
Social support score, mean (SD)	12.95 (4.11)	14.74 (4.69)	12.29 (4.22)	14.51 (4.61)
Race				
American Indian or Alaska Native	0.9 (16)	1.1 (46)	0.2 (3)	1.1 (59)
Asian	3.4 (36)	4.3 (194)	3.6 (10)	4.1 (220)
Black or African American	18.0 (166)	16.0 (446)	22.3 (72)	15.9 (540)
White	71.2 (1088)	70.5 (3429)	65.6 (355)	71.2 (4162)
Other^a^	6.5 (109)	8.2 (330)	8.2 (47)	7.7 (392)
Ethnicity				
Hispanic	24.6 (332)	26.0 (992)	26.2 (110)	25.6 (1214)
Non-Hispanic	75.4 (1093)	74.0 (3514)	73.8 (379)	74.4 (4228)
Gender				
Male	31.7 (331)	51.1 (2726)	29.3 (115)	48.2 (2008)
Female	68.3 (1084)	48.9 (1792)	70.7 (371)	51.8 (3439)
Annual household income, $				
<25 000	31.9 (373)	24.2 (681)	38.9 (166)	24.8 (888)
25 000-49 999	27.2 (327)	24.0 (835)	25.4 (111)	24.7 (1051)
50 000-74 999	15.4 (242)	17.7 (734)	15.2 (70)	17.4 (906)
75 000-99 999	8.3 (151)	12.1 (602)	5.5 (37)	11.7 (716)
≥100 000	17.3 (305)	22.0 (1429)	15.0 (93)	21.4 (1641)
Education				
High school graduate or less	32.6 (214)	36.4 (668)	27.9 (80)	35.3 (802)
Some college or technical training	39.6 (519)	31.8 (1245)	40.2 (193)	33.0 (1571)
College graduate	17.9 (360)	19.8 (1276)	14.2 (114)	19.9 (1522)
Graduate work	10.0 (333)	12.0 (1332)	7.7 (102)	11.9 (1563)
PTEs				
PSA	5.0 (71)	4.7 (211)	3.4 (17)	4.9 (265)
Other PTEs	24.9 (351)	43.1 (2045)	17.5 (83)	40.9 (2313)
Both PSA and other PTEs	64.1 (895)	29.8 (1340)	76.5 (370)	34.2 (1865)
No history of PTE	6.0 (108)	22.3 (932)	2.6 (18)	20.0 (1022)
Exposure to MVI				
Yes	25.3 (382)	16.4 (872)	30.6 (144)	17.4 (1110)
No	74.7 (1041)	83.6 (3614)	69.4 (343)	82.6 (4312)

Abbreviations: MVI, mass violence incident; PSA, physical or sexual assault; PTE, potentially traumatic event; PTSD, posttraumatic stress disorder.

^a^ Other racial subcategories included participants who reported they were biracial, Jamaican, Caribbean, Taino, North African, Middle Eastern, or Jewish.

### Logistic Regression Analyses Examining PTSD Prevalence Risk

We simultaneously entered the factors potentially associated with PTSD into logistic regression analyses (Table 4). Results indicated that younger age, female gender; lower income; experiencing either physical or sexual assault, other PTEs, or both physical or sexual assault and other PTEs; lower levels of social support; and high exposure to the MVI were each uniquely associated with both past-year and current PTSD. Specifically, for past-year PTSD, being female (odds ratio [OR], 2.32; 95% CI, 2.01-2.68), experiencing a history of both physical or sexual assault and other PTEs (OR, 9.68; 95% CI, 7.48-12.52), and high levels of exposure to the MVI (OR, 1.66; 95% CI, 1.40-1.96) were associated with the largest proportions of explained variance. Similarly, for current PTSD, being female (OR, 2.20; 95% CI, 1.77-2.73), experiencing a history of both physical or sexual assault and other PTEs (OR, 16.54; 95% CI, 9.53-28.72), and high levels of exposure to the MVI (OR, 1.82; 95% CI, 1.45-2.28) were associated with the greatest relative risk for current PTSD. Sensitivity analyses examining the regression models within each community indicated that female gender, experiencing a history of both physical or sexual assault and other PTEs, and social support were associated with PTSD across each community.

**Table 4.** Logistic Regression Analysis Examining Past-Year PTSD Prevalence

Variable	OR (95% CI)	
	Past-year PTSD (0 = no PTSD, 1 = PTSD)^a^	Current PTSD (0 = no PTSD, 1 = PTSD)^b^
Age	0.97 (0.96-0.97)	0.97 (0.96-0.98
Race		
American Indian or Alaska Native	0.45 (0.26-0.93)	0.11 (0.02-0.67
Asian	0.52 (0.34-0.78)	0.38 (0.17-0.81
Black or African American	0.77 (0.64-0.94)	1.07 (0.82-1.39
White	1 [Reference]	1 [Reference]
Other^c^	0.62 (0.47-0.83)	0.95 (0.65-1.39)
Ethnicity (1 = Hispanic, 2 = non-Hispanic)	1.43 (1.20-1.71)	1.30 (1.01-1.68)
Gender (0 = male, 1 = female)	2.32 (2.01-2.68)	2.20 (1.77-2.73)
Income	0.93 (0.88-0.98)	0.91 (0.84-0.99)
Education	1.04 (0.96-1.13)	0.93 (0.83-1.05
PTEs		
No history of PTE	1 [Reference]	1 [Reference]
PSA	4.09 (2.81-5.95)	4.82 (2.33-10.00)
Other PTE	2.61 (2.00-3.41)	3.66 (2.05-6.53)
Both PSA and other PTE	9.68 (7.48-12.52)	16.54 (9.53-28.72)
Social support	0.91 (0.90-0.93)	0.91 (0.89-0.93)
Latency (mo since MVI)	0.99 (0.99-1.00)	1.00 (0.99-1.01)
Exposure to MVI (0 = no, 1 = yes)	1.66 (1.40-1.96)	1.82 (1.45-2.28)

Abbreviations: MVI, mass violence incident; OR, odds ratio; PTE, potentially traumatic event; PSA, physical or sexual assault; PTSD, posttraumatic stress disorder.

^a^ For past-year PTSD, χ^2^_15_ = 1138.89; *P* < .001; Nagelkerke *R^2^* = 0.28; correct classification of 79% of cases.

^b^ For current PTSD, χ^2^_15_ = 635.43; *P* < .001; Nagelkerke *R^2^* = 0.24; correct classification of 91% of cases.

^c^ Other racial subcategories included participants who reported they were biracial, Jamaican, Caribbean, Taino, North African, Middle Eastern, or Jewish.

## Discussion

The findings of this survey study confirm a high prevalence of exposure to PTEs, PTSD, and risk factors for PTSD among communities affected by MVIs. Using the diagnostic criteria for PTSD, nearly one-fifth (19.0%) of community members had experienced a Criterion A level of exposure to the MVI (ie, either they or a close friend and/or family member was on site during the shooting). Regarding PTSD prevalence, approximately 1 in 4 individuals met criteria for past-year and 1 in 10 for current PTSD diagnosis, far exceeding past-year PTSD prevalence of 4.7% among US adults.^16,19^ Rates of current PTSD were lower than rates of past-year PTSD, which is consistent with research showing rates of PTSD decrease over time for several reasons including normal recovery, improvement in a sufficient number of symptoms to no longer meet full criteria, or receiving successful treatment.^20^ Among studies of direct survivors of MVIs, PTSD prevalence rates range from 9% to 91%^1^; our findings provide important perspective on the broader impact of MVIs measured by PTSD among members of the larger community.

The present findings also have important implications for identifying those at most risk for developing PTSD following MVIs. Specifically, we found that individuals who are younger, are female, have low social support, have experienced a higher number of PTEs, and have a history of physical or sexual assault were more likely to have PTSD. These findings are consistent with literature on PTSD risk factors in the context of PTEs,^19^ including MVIs.^21^ Decades of research have found that female individuals are more vulnerable to PTSD than male individuals.^22^ Reasons for this remain unclear, but it may be due to female individuals having higher rates of physical and sexual assault, greater vulnerability to the impact of stressors for biological or psychosocial reasons, or a greater willingness than male individuals to disclose symptoms. Our exploratory analyses highlight the importance of assessing for a history of PTEs—particularly physical or sexual assault—among community members affected by MVIs. Overall, our findings suggest that future response and recovery initiatives should assess for sociodemographic characteristics, history of PTEs, and access to social support to allocate resources to those individuals most at risk for experiencing PTSD in communities affected by MVIs.

The degree of exposure to the MVI plays an important role in PTSD risk. Although there is much variation in the course of PTSD,^23^ prevailing evidence suggests that many individuals who initially meet the criteria for PTSD diagnoses report some degree of natural recovery over time. Nevertheless, given the high prevalence of PTSD and mean latency in our sample of 18.94 months relative to other studies,^8^ the elapsed time since the MVI may play a less prominent role for a variety of reasons, such as the recent increase in MVI occurrence and corresponding media coverage.^2,24^ This further accords with longitudinal studies that have found exposure to intentional trauma (ie, events that involve deliberate actions to inflict harm) not only produces a more enduring course of PTSD, but may be associated with increased prevalence of PTSD over time.^25^

It is also important to consider that the rates of community PTSD may be influenced by unique features of each MVI and vulnerabilities inherent to the community. Although the majority of PTSD cases occurred in those with high exposure to the MVI, many respondents with no direct exposure met the criteria for past-year (21.0%) and current (8.9%) PTSD. Community-level factors may be especially important to consider for MVI sites with an extensive history of community violence.^26^ Indeed, important heterogeneity existed between sites with respect to ethnicity, income, time since the MVI, and degrees of exposure. Future research should consider the impact community-level differences, such as racial injustice and history of community violence, may have on rates of PTSD.

### Limitations

The present findings contribute important insight into the PTSD disease burden, as well as heightened risk for PTSD among female individuals with prior PTEs,^14^ but there are some limitations. We assessed PTSD with a highly structured, well-validated, self-reported measure. However, because we did not confirm PTSD diagnoses with clinician-administered interviews that are commonly considered the criterion standard for mental health diagnoses, caution should be used when interpreting prevalence estimates. An additional limitation relates to our address-based sampling method. This is a well-accepted, widely used method in epidemiological research that enabled us to mail invitations to 110 289 addresses in 6 communities that had experienced MVI, but there was no way for us to determine the number of vacant buildings, how many households had eligible respondents, the number of individuals who opened letters, or the number who read invitations but declined to participate. Given these limitations, our overall response rate was less than 10%, raising the possibility of nonresponse bias. However, the data were weighted to correct for nonresponse; a high percentage of community members who accessed the survey and read consent documents agreed to participate and completed the online survey, and our response rate does not depart drastically from recent large-scale community surveys assessing PTSD.^11^ Another limitation is that only a small number of participants reported they were personally on site during the MVI. This reflects the reality that only a small proportion of the population of large communities are actually present at MVIs, but it precludes analyses specifically comparing those who were on site vs those who were not.

Another limitation is that we cannot draw causal inferences from cross-sectional studies. This means that it is not possible to determine with certainty the causal sequence of when PTSD symptoms developed or whether they resulted exclusively from MVI exposure vs from a combination of factors, including exposure to the MVI, another PTE, or other factors associated with demographic characteristics or aspects of the community and related anxiety symptoms, especially for female individuals with higher rates of PTEs and PTSD. Although this study used a cross-sectional design and, therefore, causal attributions should be cautioned, the assessment of PTSD was in direct reference to the MVI, and significant associations were found between exposure to MVI and PTSD prevalence, both of which strengthen our confidence in our findings. Nevertheless, longitudinal analyses that include pre-MVI and post-MVI data points would be ideal to account for the influence of preexisting psychopathology on PTSD.

## Conclusions

The prevalence of MVIs in the US has garnered widespread media attention and captured the public’s consciousness. High rates of presumptive past-year and current PTSD were found among individuals residing in 6 communities affected by an MVI within the past decade, which portends possible enduring negative consequences for these communities. Rates of PTSD were associated with a variety of individual and MVI-specific factors, such as a history of physical or sexual abuse and degree of exposure to the MVI. These findings should be leveraged to inform response and recovery efforts in the aftermath of a future MVI in order to efficiently and accurately identify those most vulnerable to adverse mental health consequences.

## Notes

Supplement 1. **eAppendix 1.** Additional details on the assessment of posttraumatic stress disorder (PTSD) **eAppendix 2.** Additional details on the sampling strategy, data collection method, and weighting **eTable 1.** Response rate and mode of survey completion across communities **eFigure 1.** STROBE flowchart for sample selection **eTable 2.** Comparison of benchmark population parameters to sample distribution **eFigure 2.** Prevalence of PTSD across community **eTable 3.** Demographic characteristics across PTSD prevalence
eReferences

Supplement 2.
Data Sharing Statement
