# Supplementary material for: Posttraumatic Stress Disorder Among Adults in Communities With Mass Violence Incidents
Source: JAMA Netw Open. 2024 Jul 26;7(7):e2423539. doi: 10.1001/jamanetworkopen.2024.23539 (PMC11282439; doi:10.1001/jamanetworkopen.2024.23539)
Supplement: Supplement 1. — eAppendix 1. Additional details on the assessment of posttraumatic stress disorder (PTSD) eAppendix 2. Additional details on the sampling strategy, data collection method, and weighting eTable 1. Response rate and mode of survey completion across communities eFigure 1. STROBE flowchart for sample selection eTable 2. Comparison of benchmark population parameters to sample distribution eFigure 2. Prevalence of PTSD across community eTable 3. Demographic characteristics across PTSD prevalence eReferences [file jamanetwopen-e2423539-s001.pdf]

Supplemental Online Content

Moreland AD, Rancher C, Davies F, et al. Posttraumatic stress disorder among adults in communities with mass violence incidents. *JAMA Netw Open*. 2024;7(7):e2423539. doi:10.1001/jamanetworkopen.2024.23539

eAppendix 1. Additional details on the assessment of posttraumatic stress disorder (PTSD)
eAppendix 2. Additional details on the sampling strategy, data collection method, and weighting
eTable 1. Response rate and mode of survey completion across communities
eFigure 1. STROBE flowchart for sample selection
eTable 2. Comparison of benchmark population parameters to sample distribution
eFigure 2. Prevalence of PTSD across community
eTable 3. Demographic characteristics across PTSD prevalence
eReferences

This supplemental material has been provided by the authors to give readers additional information about their work.

© 2024 Moreland AD et al. *JAMA Network Open.*

eAppendix 1. Additional details on the assessment of posttraumatic stress disorder (PTSD).

Assessment of Posttraumatic Stress Disorder (PTSD)

PTSD was measured using the National Stressful Events Survey (NSES) PTSD measure[1] developed in conjunction with the American Psychiatric Association (APA) Diagnostic and Statistical Manual of Mental Disorders-5 (DSM-5) PTSD Workgroup.[2] The NSES PTSD measure is a self-assessment survey designed to mimic a highly structured clinical interview that follows DSM-5 PTSD diagnostic criteria including that the symptoms produce either substantial distress or impaired functioning.

The first part of the module includes an assessment of potentially traumatic events (PTE), described below. The purpose of assessing for PTEs is to prime the respondent to think about exposure to these PTEs in reference to the PTSD symptoms. The NSES PTSD measure includes 20 questions assessing each DSM-5 PTSD symptom as well as two items assessing whether PTSD symptoms resulted in significant distress or impairment. Respondents are asked about how recently they have experienced each symptom and how much distress it has caused them (e.g., symptom severity).

Consistent with other structured self-report measures of PTSD (Grasso et al., 2015), scoring algorithms can be used to identify respondents who are likely to qualify for a diagnosis of PTSD based on their responses. Specifically, respondents must endorse experiencing symptom-based criteria for each PTSD symptom cluster, including: intrusion symptoms (PTSD Criterion B; American Psychiatric Association, 2013) by reporting at least 1 reexperiencing symptom (e.g., "Have you had repeated, distressing memories about a stressful experience from the past that just popped into your head when you weren't expecting it or when something reminded you of the stressful experience?"); at least 1 avoidance symptom (PTSD Criterion C;

© 2024 Moreland AD et al. *JAMA Network Open.*

American Psychiatric Association, 2013; (e.g., “Have you tried to avoid having thoughts, feelings, or physical sensations that reminded you of a stressful event/experience that happened to you?”); at least 2 symptoms reflecting negative changes in mood and cognitions (PTSD Criterion D; American Psychiatric Association, 2013; e.g., “Sometimes people are not able to remember an important aspect of a stressful experience. Did this ever happen to you after a stressful experience?”); at least 2 symptoms reflecting alterations in arousal and reactivity (PTSD Criterion E; American Psychiatric Association, 2013; e.g., “Have you felt jumpy or easily startled when you heard an unexpected noise?”); and significant distress or impairment (PTSD Criterion G; American Psychiatric Association, 2013) .by reporting at least 1 item (e.g., “When you had these bad moods, feelings, and memories, did they ever affect your work or school performance, your relations with your family or friends, or your ability to take care of things in your personal life?”). If these criteria are all met, diagnosis of PTSD is considered likely (Grasso et al., 2015). In the current study respondents were considered to have met criteria for current PTSD if they endorsed experiencing each of these symptoms “within the past month” (PTSD Criteria F; American Psychiatric Association, 2013) and were considered to have met for past year PTSD if they endorsed experiencing these symptoms “within the past month,” “within the past 6 months,” or “within the past year.” The NSES PTSD measure also includes a measure of symptom severity, which was not included in the present study.

The following instructions were provided to participants:

“Many people develop problems or difficulties after extremely stressful events/experiences such as the ones we just asked you about or the shooting. Please tell us if you have EVER had any of the following problems, including if you have the problem currently”. Here are verbatim examples of several of the actual PTSD questions used in the survey:

36. You had repeated, distressing memories about a stressful experience from the past that just popped into your head when you weren’t expecting it or when something reminded you of the stressful experience?

© 2024 Moreland AD et al. *JAMA Network Open.*

1 Yes [CONTINUE]
2 No [SKIP TO Q37]
8 Don’t know/Not sure [SKIP TO Q37] 9 I prefer not to answer [SKIP TO Q37]]

36A. When is the last time one of these distressing memories popped into your head?
1 Within the past month [CONTINUE]
2 Within the past 6 months [SKIP TO Q37]
3 Within the past year [SKIP TO Q37]
4 More than 1 year ago [SKIP TO Q37] 8 Don’t know/Not sure [SKIP TO Q37]
9 I prefer not to answer

36B. How much have you been bothered by these distressing memories during the past month?
1 Not at all
2 A little bit
3 Moderately
4 Quite a bit
5 Extremely
8 Don’t know/Not sure 9 I prefer not to answer

38. You had “flashbacks,” that is, you suddenly acted or felt as if a stressful experience from the past was happening all over again?
1 Yes [CONTINUE]
2 No [SKIP TO Q39]
8 Don’t know/Not sure [SKIP TO Q39] 9 I prefer not to answer
[Same follow up questions as above]

41. You tried to avoid having thoughts, feelings, or physical sensations that reminded you of a stressful event/experience that happened to you?
1 Yes [CONTINUE]
2 No [SKIP TO Q42]
8 Don’t know/Not sure [SKIP TO Q42] 9 I prefer not to answer [SKIP TO Q42]
[Same follow up questions as above]

42. You went out of your way to avoid people, places, activities, conversations, objects, or situations that reminded you of a stressful experience?
1 Yes [CONTINUE]
2 No [SKIP TO Q43]
8 Don’t know/Not sure [SKIP TO Q43] 9 I prefer not to answer [SKIP TO Q43] 42A.
[Same follow up questions as above]

43. Sometimes people are not able to remember an important aspect of a stressful experience. Did this ever happen to you after a stressful experience?
1 Yes [CONTINUE]
2 No [SKIP TO Q44]
8 Don’t know/Not sure [SKIP TO Q44] 9 I prefer not to answer [SKIP TO Q44
[Same follow up questions as above]

45.You changed the way you thought about yourself or about the future after a stressful event/experience? That is, you thought that you were a really bad person, that you could never trust anyone again, that nothing good could ever happen, that you would never be able to have a career, a good relationship, marriage, or children, or that your life would somehow be cut short?

© 2024 Moreland AD et al. *JAMA Network Open.*

1 Yes [CONTINUE]
2 No [SKIP TO Q45]
8 Don’t know/Not sure [SKIP TO Q45] 9 I prefer not to answer [SKIP TO Q45]
[Same follow up questions as above]

50. You had a period of time lasting more than a few days when you were extremely irritable or angry to the point that you yelled at other people, got into fights, or destroyed things?
1 Yes [CONTINUE]
2 No [SKIP TO Q51]
8 Don’t know/Not sure [SKIP TO Q51] 9 I prefer not to answer [SKIP TO Q51]
[Same follow up questions as above]

53. You felt jumpy or easily startled when you heard an unexpected noise?
1 Yes [CONTINUE]
2 No [SKIP TO Q54]
8 Don’t know/Not sure [SKIP TO Q54]
9 I prefer not to answer [SKIP TO Q54] [Same follow up questions as above]

PTSD Criterion G: “The disturbance causes clinically significant distress or impairment in social, occupational, or other important areas of functioning.”

56. When you experienced these problems that we have just asked you about, how distressing was it for you?
1 Not at all
2 A little bit
3 Moderately
4 Quite a bit
5 Extremely
8 Don’t know/Not sure
9 I prefer not to answer

Bad moods, bad feelings, and bad memories like those we asked you about can sometimes affect your life in other ways.

57. When you had these bad moods, feelings, and memories, did they ever affect your work or school performance, your relations with your family or friends, or your ability to take care of things in your personal life?
1 Yes
2 No
8 Don’t know/Not sure
9 I prefer not to answer

Validity and reliability of the PTSD assessment module

The modified NSES PTSD measure is not a “gold standard” clinician administered semi-structured interview such as the Structured Clinical Interview for DSM-5 PTSD Module (SCID-5)[3] or the Clinician-Administered PTSD Scale for DSM-5 (CAPS-5).[4] This raises the question of

© 2024 Moreland AD et al. *JAMA Network Open.*

whether it is sufficiently reliable and valid to produce acceptably accurate estimates of presumptive PTSD in epidemiological studies of communities that have experienced natural or manmade disasters. To address this question, we provide the following information. First, the internal consistency for the 20 PTSD symptoms was .91 which is classified as excellent. Second, data from the DSM-IV PTSD Field Trial[5] using the DSM-IV form of our module found good agreement between PTSD diagnoses obtained by lay interviewers using our module and PTSD diagnoses by mental health professionals using the semi-structured SCID DSM-IV PTSD Module (kappa = .77), confirming that our measure is reliable when compared to a "gold standard" clinician interview. Finally, the NSES PTSD measure was used in a DSM-5 PTSD work group commissioned study[1] and produced a national U.S. past-year DSM-5 PTSD prevalence estimate of 4.7%. A large national study that assessed PTSD using DSM-5 criteria with face-to-face in-person highly structured interviews replicated our findings exactly (i.e., the past year prevalence was 4.7%).[6] Both estimates were cited in the APA's DSM-5-TR Manual as national prevalence estimates for PTSD. These data suggest that the NSES PTSD measure has sufficient reliability, validity, and acceptance in the PTSD field to produce justifiable estimates of presumptive PTSD in post-disaster studies.

Social Support

Social support was measured via a modified five-item version of the Medical Outcomes Study module that assesses social support over the past six months. Respondents were asked how often they had someone available to "help you if you were confined to bed", "give good advice about a crisis", "get together with for relaxation, "confide in or talk with about your problems", and "love you and make you feel wanted". Response options ranged from "none of the time" (score=1) to "all of the time" (score=4) (scale range 5-20). This scale has good reliability

(α=0.86). Based on results from prior studies of the 9/11 terrorist attacks[7] and hurricanes in Florida[8], we defined low social support as a score of ≤15. 70.7% of respondents reported low social support.

© 2024 Moreland AD et al. *JAMA Network Open.*

eAppendix 2. Additional details on the sampling strategy, data collection method, and weighting.

Sampling Strategy and Data Collection Method

The goal of our sampling strategy was to locate household probability samples of adults (age 18 and older) who had potential exposure to each MVI in their respective communities. The three major household probability sampling options we considered were: 1) in person sampling of households; 2) dual-frame (i.e., landline and mobile telephone) random digit dialing (RDD) sampling in which phone numbers are called randomly to identify individuals who reside in households within the geographic area; and 3) address-based sampling (ABS) in which letters describing the study are mailed to randomly selected households within the specified geographic area. Each method has advantages and disadvantages.

In person sampling surveys were once considered to be state of the art and were widely used. However, they are very expensive and cost 3-4 times as much as RDD dual-frame or ABS surveys. When 98% of US households had landlines, RDD sampling became a widely used, viable option. However, the proportion of households with landline phones has dropped dramatically over the past twenty years, necessitating use of RDD dual-frame sampling including mobile phones. RDD dual-frame sampling surveys have much lower response rates than previous landline-only RDD surveys. Consequently, ABS sampling surveys, which are comparable in cost to RDD dual-frame surveys but much less expensive than in-person sampling surveys, are now considered to be a first-line option for household surveys, particularly when there is a need to sample households in a specified geographic area.

Given budgetary constraints and the need to limit the sampling frame to households in each respective community that had experienced an MVI, we elected to use ABS sampling. The

© 2024 Moreland AD et al. *JAMA Network Open.*

target population for the project was adults aged 18 and over who lived in each respective community during the time of the MVI. The sample design included an address-based sample of addresses in each community, drawn from the US Postal Service's Delivery Sequence File (DSF). The address-based sample was provided by Marketing Systems Group and consisted of a random sample of addresses from each community.

Five basic methods are used to collect data in household surveys: 1) face-to-face in person interviews; 2) face-to-face interviews using computer-administered modules for sensitive content items (e.g., respondents are given a tablet with earphones by the interviewer in which there is computerized administration of the survey; instructions and questions are recorded and read out loud to respondents who then key in their answers); 3) telephone interviews: 4) paper surveys that are mailed to respondents; and 5) web-based, self-administered surveys that are completed by respondents online. The first three of these methods are primarily used in the context of in-person or telephone surveys, but ABS surveys typically use one or both of the paper survey and web-based survey methods. The most commonly used method in ABS surveys is push-to-web with a follow-up in which paper surveys are mailed to those who do not respond to requests to complete the web-based survey. We decided to use this push-to-web with mailed paper survey follow-up data collection method in these six communities.

Another justification for using these two data collection modes (web-surveys with follow-up paper surveys) is that the community survey contains many questions on sensitive topics (e.g., potentially traumatic event exposure, alcohol and drug use, mental health problems), and there is considerable research showing that surveys collecting data using live interviewers often obtain lower and less accurate reports of experiences that are sensitive, embarrassing, or stigmatizing than when the same survey does not include a face-to-face or telephone interviewer.

© 2024 Moreland AD et al. *JAMA Network Open.*

Neither web or paper surveys require contact with a live interviewer, and studies comparing results using paper vs web surveys have not found meaningful differences using these two modes.

All addresses were mailed information about the project, a URL directing them to the online survey, and a unique project ID number. All addresses were also sent a second mailing which consisted of a postcard thanking those that have completed and reminding those that have not. The last mailing included a paper copy of the needs assessment and a business reply envelope (BRE). The mailing was sent to address based participants who had not responded to the initial invitation to complete the online needs assessment and also excluded any undeliverable addresses from previous mailings.

The description of the study in the recruitment materials was the following: “This project is designed to find out what effects the [2019 shooting in the Oregon Historic District of Dayton, Ohio] had on people in your community. To find out how the community has been affected, we are sending invitations to people who have been selected randomly from the entire community.”

Specific details on the numbers of mailings sent at each stage were not provided; however, the overall response rate and mode of survey completion is depicted below in eTable1. A total of 110,289 addresses were mailed recruitment letters. There is no way to determine how many households were ineligible, opened the letter and passively declined, or if the letter was sent to a vacant address. We can determine that 6,867 participants opened the letter, read the instructions, and accessed the survey. Of these, 5,991 (87.2%) completed the survey. There were 432 (6.3%) partial completions and 443 (6.5%) screen-outs. Of the complete surveys, 5,389 (78%) were web surveys and 1,478 (21.5%) were paper surveys.

© 2024 Moreland AD et al. *JAMA Network Open.*

eTable1
Response rate and mode of survey completion across communities

Community	Number of Mailings Sent	Respondents	Web Survey	Paper Survey
Dayton	19,801	1,144	78%	22%
El Paso	19,596	1,139	78%	22%
Parkland	24,381	1,075	84%	16%
Pittsburgh	20,401	1,145	79%	21%
San Bernadino	10,000	393	68%	32%
Virginia Beach	16,110	1,095	75%	25%
**Total**	110,289	5,991	78% (4,694)	22% (1,297)

eFigure 1. STROBE flowchart for sample selection

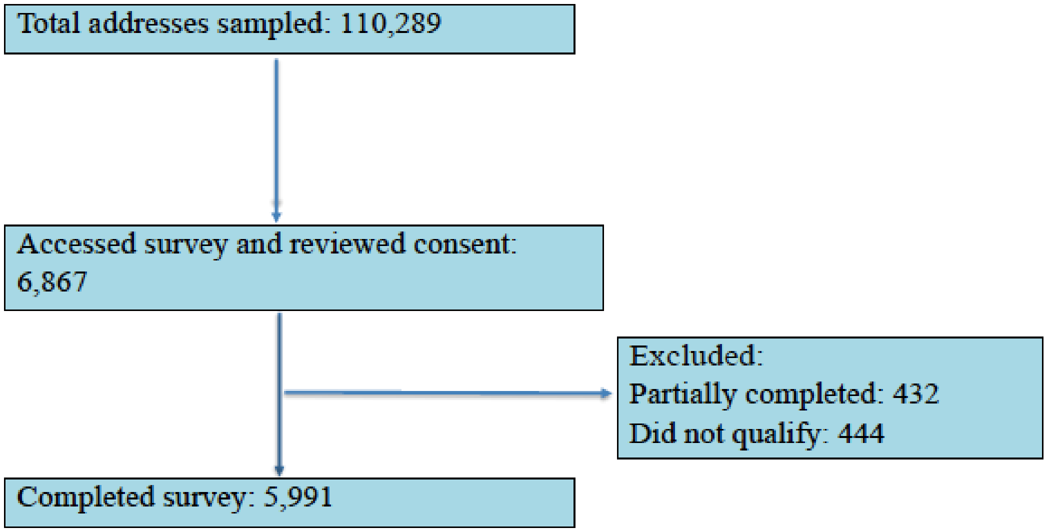

<u>Weighting</u>

To the extent that those responding to the survey are different from those who did not, there is a risk that estimates could be subject to nonresponse bias. Nonresponse weights based on propensity score adjustments were computed to adjust for differential nonresponse to the survey. The sample frame provided by Market Systems Group includes variables appended to each address that describe the housing unit as well as demographic characteristics of the occupant(s). These variables were used to predict the likelihood that a household associated with the address would respond to the survey.

© 2024 Moreland AD et al. *JAMA Network Open.*

A logistic regression model was estimated in which responding to the survey was regressed on the following flags provided by MSG based on proprietary marketing data (we cannot treat them as true values, but they are helpful in creating response propensity models): 1); Zip Code; 2) Dwelling type (single vs. multi-family); 3) Tenure of the housing unit (rented vs. owned); 4) Presence of a landline telephone in the housing unit; 5) Presence of a child in the household; 6) Number of adults in the household; 7) Sex; 8) Education level; 9) Race (Black/African American or not); 10) Marital status; and 11) Income.

The response propensity model was estimated using all sampled addresses, excluding cases known to be ineligible, such as vacant and undeliverable addresses and screen-outs. The estimated propensities were used to divide cases into approximately equal size groups using the quartiles of the estimated propensity score. The nonresponse weight for respondents was computed as the inverse of the response rate in each quartile. This approach helps to protect against model misspecification, relative to using the inverse of the response propensities.

The final baseweight for each respondent was computed as the product of the household size adjustment and the nonresponse weight. In the final stage of weighting, the final baseweights for respondents were calibrated to population parameters using raking, or iterative proportional fitting. The characteristics of respondents were aligned to match population benchmarks on sex, education level, race/Hispanic ethnicity, marital status, and household size. The weighting parameters were obtained from an analysis of the Census Bureau's 2018 American Community Survey (ACS) one-year estimates, filtered on aged 18 years and older residing in households in each of the six communities. The eTable2 below compares the unweighted and the weighted sample distributions to population parameters.

© 2024 Moreland AD et al. *JAMA Network Open.*

eTable 2<br>*Comparison of benchmark population parameters to sample distribution*			
**DAYTON**	**Benchmark**	**Weighted**	**Unweighted**
Male 18-29	12.3%	12.0%	4.0%
Male 30-44	11.5%	10.9%	8.6%
Male 45-64	16.0%	15.2%	12.8%
Male 65+	7.7%	7.3%	5.9%
Female 18-29	12.4%	13.1%	10.9%
Female 30-44	11.8%	12.1%	19.2%
Female 45-64	17.1%	17.6%	26.5%
Female 65+	11.3%	11.7%	12.1%
White only Non-Hispanic	66.4%	66.4%	69.1%
Black only Non-Hispanic	27.5%	27.4%	22.7%
All others	6.1%	6.1%	8.1%
**EL PASO**	**Benchmark**	**Weighted**	**Unweighted**
Male 18-29	13.6%	13.6%	3.5%
Male 30-44	13.1%	13.1%	8.1%
Male 45-64	14.0%	14.0%	14.7%
Male 65+	7.4%	7.4%	9.7%
Female 18-29	12.6%	12.6%	10.2%
Female 30-44	13.0%	13.0%	18.4%
Female 45-64	16.2%	16.2%	25.6%
Female 65+	10.2%	10.2%	10.0%
White only Non-Hispanic	13.8%	13.8%	16.5%
Black/African American only Non-Hispanic	3.2%	3.2%	1.9%
Hispanic	80.4%	80.4%	78.6%
Other race/Multi-race Non-Hispanic	2.7%	2.7%	3.0%
**PARKLAND**	**Benchmark**	**Weighted**	**Unweighted**
Stratum 1: Male 18-29	5.0%	5.0%	3.2%
Stratum 1: Male 30-44	6.7%	6.7%	4.8%
Stratum 1: Male 45-64	12.4%	12.4%	15.8%
Stratum 1: Male 65+	4.9%	4.9%	8.7%
Stratum 1: Female 18-29	3.9%	3.9%	6.5%
Stratum 1:Female 30-44	8.1%	8.1%	11.3%
Stratum 1: Female 45-64	12.3%	12.4%	28.5%
Stratum 1: Female 65+	5.4%	5.4%	8.1%
Stratum 2: Male 18+	19.4%	19.4%	4.6%
Stratum 2: Female 18-44	10.0%	10.0%	3.9%
Stratum 2: Female 45+	12.0%	12.0%	4.7%
Stratum 1: White only Non-Hispanic	38.6%	38.6%	60.4%

© 2024 Moreland AD et al. *JAMA Network Open.*

Stratum 1: Black only Non-Hispanic	6.1%	6.1%	3.7%
Stratum 1: Hispanic	12.0%	12.0%	14.6%
Stratum 1: Other/Multi-race Non-Hispanic	1.9%	1.9%	8.2%
Stratum 2: White only Non-Hispanic	21.6%	21.6%	7.8%
Stratum 2: All others	19.7%	19.8%	5.3%
**PITTSBURGH**	**Benchmark**	**Weighted**	**Unweighted**
Male 18-29	14.5%	14.5%	7.8%
Male 30-44	11.3%	11.3%	8.0%
Male 45-64	13.6%	13.6%	8.6%
Male 65+	8.1%	8.1%	10.6%
Female 18-29	14.7%	14.7%	15.0%
Female 30-44	11.1%	11.1%	17.6%
Female 45-64	15.1%	15.1%	17.6%
Female 65+	11.7%	11.7%	14.8%
White only Non-Hispanic	70.0%	70.0%	78.5%
Black only Non-Hispanic	21.8%	21.8%	8.5%
Asian only Non-Hispanic	2.3%	2.4%	2.3%
Hispanic	3.6%	3.6%	5.8%
Other/multi-race Non-Hispanic	2.3%	2.3%	5.0%
**SAN BERNARDINO**	**Benchmark**	**Weighted**	**Unweighted**
Total 18-29	28.3%	28.2%	14.3%
Male 30-44	13.9%	13.9%	5.6%
Male 45-64	14.1%	14.1%	11.7%
Male 65+	5.9%	5.9%	11.2%
Female 30-44	14.2%	14.2%	19.1%
Female 45-64	15.3%	15.3%	26.0%
Female 65+	8.3%	8.3%	12.2%
White Non-Hispanic	20.7%	20.7%	30.0%
Black Non-Hispanic	10.7%	10.7%	13.7%
Hispanic	61.0%	61.0%	45.0%
Other/Multi-race Non-Hispanic	7.6%	7.6%	11.2%
**VIRGINIA BEACH**	**Benchmark**	**Weighted**	**Unweighted**
Total 18-29	23.4%	23.1%	5.1%
Male 30-44	14.4%	14.6%	6.1%
Male 45-64	16.1%	16.2%	18.6%
Male 65+	6.1%	6.1%	13.3%
Female 30-44	14.6%	14.6%	15.4%
Female 45-64	17.9%	17.9%	27.2%
Female 65+	7.5%	7.6%	14.2%
White only Non-Hispanic	62.6%	62.5%	76.8%

© 2024 Moreland AD et al. *JAMA Network Open.*

Black only Non-Hispanic	18.3%	18.2%	10.1%
Hispanic	7.7%	7.6%	3.5%
Asian only Non-Hispanic	9.4%	9.4%	4.4%
Other/Multi-race Non-Hispanic	2.0%	2.3%	5.3%

eFigure 2

Prevalence of PTSD across community

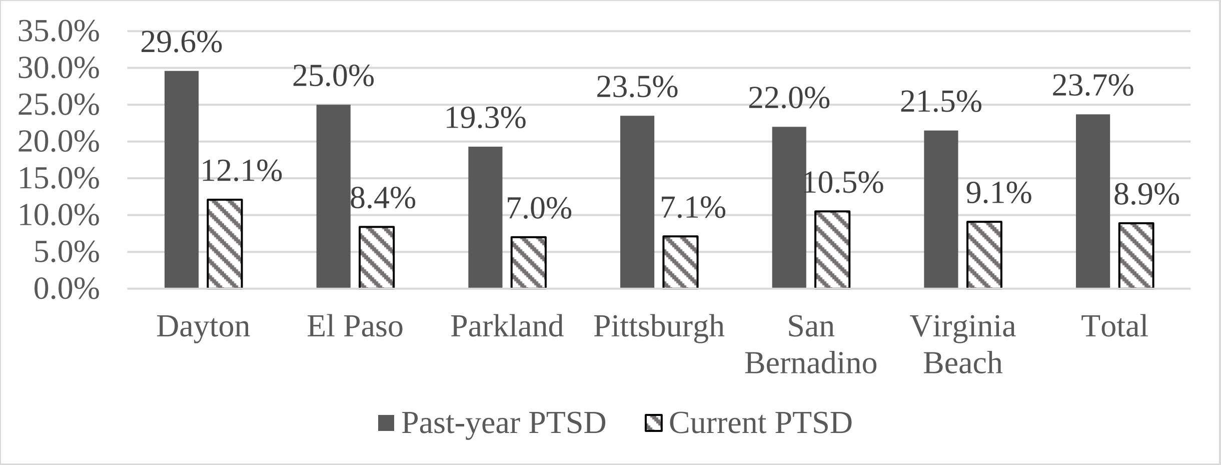

Note. Weighted comparisons indicated significant differences across community in the prevalence of past-year, X^2 (5) = 37.75, $p < .001$, and current, X^2 (5) = 25.19, $p < .001$, PTSD

© 2024 Moreland AD et al. *JAMA Network Open.*

eTable 3. Demographic characteristics across PTSD prevalence

	Past-year PTSD (1,417)			Current PTSD (530)		
	M (SD)			M (SD)		
**Age**	39.89 (15.18)			39.41 (14.82)		
**Latency (months since MVI)**	18.27 (11.24)			18.72 (12.16)		
**Social support**	12.95 (4.11)			12.29 (4.22)		
	N	Unweighted %	Weighted %	N	Unweighted %	Weighted %
Race						
White	1088	24.1%	24.1%	355	7.9%	8.3%
Black or African American	166	27.1%	26.2%	72	11.8%	12.1%
American Indian or Alaskan Native	16	25.8%	19.7%	3	4.8%	1.7%
Asian	36	15.7%	20.1%	10	4.3%	7.9%
Other and Multi Racial	109	24.8%	20.0%	47	10.7%	9.5%
Ethnicity						
Hispanic	332	25.1%	22.9%	110	8.3%	9.1%
Non-Hispanic	1093	23.7%	24.2%	379	8.2%	8.9%
Gender						
Male	331	15.6%	16.0%	115	5.4%	5.6%
Female	1084	28.5%	30.1%	371	9.7%	11.8%
Income						
Less than $25,000	373	35.4%	29.8%	166	15.7%	13.3%
$25,000 to $49,999	327	28.1%	26.7%	111	9.6%	9.2%
$50,000 to $74,999	242	24.8%	21.8%	70	7.2%	7.9%
$75,000 to $99,999	151	20.1%	18.0%	37	4.9%	4.4%
$100,000 or more	305	17.6%	20.2%	93	5.4%	6.5%
Education						
High School graduate or less	214	24.3%	21.8%	80	9.1%	9.5%
Some college or technical training	519	29.4%	28.0%	183	10.9%	10.6%
College graduate	360	22.0%	21.9%	114	7.0%	6.5%
Graduate work	333	20.0%	20.6%	102	6.1%	6.0%
Potentially traumatic events (PTEs)						
Physical or sexual assault (PSA)	71	25.2%	24.9%	17	6.0%	6.3%
Other PTEs	351	14.6%	15.3%	83	3.5%	4.0%
Both PSA and other PTEs	895	40.0%	40.1%	370	16.6%	17.9%
No history of PTE	108	10.4%	7.7%	18	1.7%	1.3%
Exposure to MVI						
Yes	382	30.5%	32.7%	144	11.5%	14.8%
No	1041	22.4%	22.0%	343	7.4%	7.6%

Note. PTSD = Posttraumatic Stress Disorder; MVI = Mass Violence Incident
Statistically significant weighted differences in past year PTSD are **bolded** and differences in current PTSD are *italicized, ps* < .05

© 2024 Moreland AD et al. *JAMA Network Open.*

eReferences

1. Kilpatrick DG, Resnick HS, Milanak ME, Miller MW, Keyes KM, Friedman MJ. National estimates of exposure to traumatic events and PTSD prevalence using DSM-IV and DSM-5 criteria. J Trauma Stress. 2013;26(5):537-547. doi:10.1002/jts.21848.
2. American Psychiatric Association. Diagnostic and Statistical Manual of Mental Disorders. 5th ed. American Psychiatric Association; 2013.
3. First MB, Williams JBW, Karg RS, Spitzer RL. User’s Guide for the SCID-5-CV Structured Clinical Interview for DSM-5® Disorders: Clinical Version. American Psychiatric Publishing, Inc.; 2016:xii, 158.
4. Weathers FW, Bovin MJ, Lee DJ, et al. The Clinician-Administered PTSD Scale for DSM-5 (CAPS-5): Development and initial psychometric evaluation in military veterans. Psychol Assess. 2018;30(3):383-395. doi:10.1037/pas0000486.
5. Kilpatrick DG, Resnick HS, Freedy JR, et al. The posttraumatic stress disorder field trial: evaluation of the PTSD construct—criteria A through E. In: Widiger TA, Frances AJ, Pincus HA, et al, eds. DSM-IV sourcebook. Vol 4. Washington, DC: American Psychiatric Association Press, 1998:803–44.
6. Goldstein RB, Smith SM, Chou SP, et al. The epidemiology of DSM-5 posttraumatic stress disorder in the United States: results from the National Epidemiologic Survey on Alcohol and Related Conditions-III. Soc Psychiatry Psychiatr Epidemiol. 2016;51(8):1137-1148. doi:10.1007/s00127-016-1208-5
7. Galea S, Ahern J, Resnick H, et al. Psychological sequelae of the September 11 terrorist attacks in New York City. New England Journal of Medicine. 2002;346(13):982-987. doi:10.1056/NEJMsa013404
8. Kilpatrick DG, Koenen KC, Ruggiero KJ, et al. The serotonin transporter genotype and social support and moderation of posttraumatic stress disorder and depression in hurricane-exposed adults. Am J Psychiatry. 2007;164(11):1693-1699. doi:10.1176/appi.ajp.2007.06122007

© 2024 Moreland AD et al. *JAMA Network Open.*
