# Supplementary material for: Posttraumatic Stress Disorder Among Adults in Communities With Mass Violence Incidents
Source: JAMA Netw Open. 2024 Jul 26;7(7):e2423539. doi: 10.1001/jamanetworkopen.2024.23539 (PMC11282439; doi:10.1001/jamanetworkopen.2024.23539)
Supplement: Supplement 2. — Data Sharing Statement [file jamanetwopen-e2423539-s002.pdf]

Data Sharing Statement

Moreland. Posttraumatic Stress Disorder Among Adults in Communities With Mass Violence Incidents. *JAMA Netw Open*. Published July 26, 2024.
doi:10.1001/jamanetworkopen.2024.23539

Data
Data available: No
